# Pathogenic Germline Variants in Patients With Metaplastic Breast Cancer

**DOI:** 10.1001/jamanetworkopen.2024.60312

**Published:** 2025-02-18

**Authors:** Kaitlin Demarest, Arravinth Anantharajah, Kara N. Maxwell, Mersedeh Rohanizadegan, Angela Bradbury, Katherine L. Nathanson, Anne Marie McCarthy, Susan M. Domchek, Anupma Nayak, Payal D. Shah

**Affiliations:** 1 Basser Center for BRCA, University of Pennsylvania, Philadelphia; 2 Department of Medicine, Perelman School of Medicine, Hospital of the University of Pennsylvania, Philadelphia; 3 Abramson Cancer Center, University of Pennsylvania, Philadelphia; 4 Department of Medicine, Division of Translational Medicine & Human Genetics, Perelman School of Medicine, Hospital of the University of Pennsylvania, Philadelphia; 5 Department of Biostatistics, Epidemiology, and Informatics, University of Pennsylvania, Perelman School of Medicine, Philadelphia; 6 Department of Pathology and Laboratory Medicine, Perelman School of Medicine, Hospital of the University of Pennsylvania, Philadelphia

## Abstract

**Importance:**

Metaplastic breast cancer (MpBC) is a rare, heterogeneous disease often associated with inferior outcomes. A growing body of literature describes the clinical and molecular features of MpBC, yet limited data describe the pathogenic germline variants (PGVs) in breast cancer susceptibility genes among affected individuals.

**Objective:**

To examine the frequency and types of PGVs in breast cancer genes among patients with MpBC.

**Design, Setting, and Participants:**

This is a descriptive retrospective cohort study of patients who received a diagnosis of MpBC at the University of Pennsylvania between January 2010 and May 2023. Electronic medical records were reviewed for demographic, clinicopathologic, and germline genetic testing information. Germline variant status was independently confirmed by a licensed genetic counselor and a physician with expertise in genetics. MpBC diagnosis and subtype were confirmed by a breast pathologist. Participants were identified via query of an institutional pathology database for reports signed between January 2010 and May 2023 including the term *metaplastic*. Among 320 initially obtained reports, 272 individuals had confirmed MpBC and were included in the study.

**Exposure:**

Germline genetic testing to investigate the presence of PGVs in breast cancer susceptibility genes.

**Main Outcomes and Measures:**

The primary outcome measurement was the prevalence of PGVs in breast cancer susceptibility genes among participants. The hypothesis that individuals with MpBC have an enrichment of PGVs in genes associated with inherited breast cancer risk was formulated before data collection.

**Results:**

The total sample size was 272 women, and the median age at diagnosis was 58 years (range, 20-102 years); all were biological female patients; 143 of 272 (52.6%) had documentation of germline genetic testing; and participants with testing were significantly younger than those without (median age, 53 years [range, 20-79 years] vs 63 years [range, 29-102 years]; *P* < .001). Of the 143 patients, 24 (16.8%) had a PGV in a breast cancer susceptibility gene (*BRCA1*, n = 17; *BRCA2*, n = 5; *PALB2*, n = 1; *CHEK2*, n = 1). Patients with PGV-associated MpBC received a diagnosis at a younger age than those with sporadic disease, but there were no significant differences in hormone receptor positivity, ERBB2 status, or metaplastic subtype.

**Conclusions and Relevance:**

In this cohort study of patients with MpBC, a substantial proportion of clinically tested patients had a PGV in a breast cancer susceptibility gene, most commonly *BRCA1*. Germline testing was high yield in patients with MpBC, many of whom would be included in current germline testing eligibility criteria.

## Key Points

**Question:**

What are the frequencies and types of pathogenic germline variants (PGVs) in breast cancer susceptibility genes among individuals with metaplastic breast cancer (MpBC)?

**Findings:**

In this cohort study of 143 patients with MpBC who underwent clinical germline genetic testing, 24 (16.8%) had a PGV in a breast cancer susceptibility gene, most commonly in *BRCA1*.

**Meaning:**

These findings suggest that a substantial proportion of patients with this rare breast cancer subtype may have clinically meaningful PGVs in breast cancer genes; therefore, germline testing for all patients with MpBC may be high yield.

## Teaser

This cohort study examines the frequency and types of pathogenic germline variants in breast cancer genes among patients with metaplastic breast cancer.

## Introduction

Metaplastic breast cancers (MpBCs) comprise a rare, heterogeneous group of breast cancers that demonstrate elements of squamous, mesenchymal, or neuroectodermal differentiation.^1,2,3^ These tumors represent 0.2% to 1% of primary breast cancers diagnosed in the United States^1,4,5,6^ and are often associated with an advanced stage at diagnosis, decreased sensitivity to systemic therapies,^4,6,7^ and inferior outcomes.^8,9,10^ Histologically, World Health Organization classification describes 4 aggressive subgroups of MpBC: spindle cell carcinoma, squamous cell carcinoma, MpBC with heterologous mesenchymal differentiation, and mixed MpBCs. Lower-risk histologies are less common and include low-grade adenosquamous and fibromatosis-like MpBCs.^11^ The majority of MpBCs lack estrogen receptor (ER), progesterone receptor (PR), and erb-b2 receptor tyrosine kinase 2 (ERBB2) expression.^12,13^ Several studies have examined the molecular features of this group, noting diversity in gene-expression profiling, a high frequency of acquired, somatic *TP53* variants, and alterations affecting the PI3K-AKT1-MTOR pathway, including *PIK3CA* and *PTEN* variants.^4,14,15,16,17^

Genomic features of homologous recombination deficiency, including *BRCA*-like genomic signatures, have also been noted in metaplastic breast tumors across histologic subgroups.^18,19^ One series noted *BRCA1*-like genomic profiles, based on the number of large-scale state transitions, in 31% of evaluated MpBCs.^16^ Limited data describe whether the observed *BRCA*-like molecular features in MpBC are indicative of, or explained by, a higher frequency of pathogenic germline variants (PGVs) in breast cancer predisposition genes associated with DNA repair, such as *BRCA1*, *BRCA2*, and *PALB2*.^20,21,22,23,24,25^ A recent study described a higher frequency of PGVs in *BRCA1* in MpBC compared with other breast cancer subtypes; however, this study included only 23 patients with MpBC, and the patients were ascertained from a genetic counseling and testing clinic.^26^ Although PGVs in breast cancer susceptibility genes are present in approximately 5% to 10% of individuals with breast cancer overall and approximately 12% to 15.8% of those with triple-negative breast cancer,^27,28,29,30^ the frequency of PGVs specifically in MpBC has not been described.

Understanding the types and frequency of PGVs in patients with MpBC has potential implications for therapeutics^31,32,33^ for those affected by cancer and for identification of unaffected carriers of PGVs in cancer predisposition genes. We present, to our knowledge, the largest reported dataset of patients with MpBC who underwent germline genetic testing to describe the prevalence and types of PGVs in breast cancer susceptibility genes.

## Methods

The present study was a retrospective cohort study evaluating the PGVs in breast cancer susceptibility genes in an institutional cohort of patients with MpBC. The hypothesis that individuals with MpBC have an enrichment of PGVs in genes associated with inherited breast cancer risk was formulated before data collection. The study protocol was approved by the institutional review board at the University of Pennsylvania. A US Health Insurance Portability and Accountability Act waiver of consent was obtained before the conduct of this analysis to allow for review and reporting of clinical and germline genetic test results for patients with MpBC, including patients both living and deceased, from whom consent could not feasibly be obtained. The Strengthening the Reporting of Observational Studies in Epidemiology (STROBE) reporting guideline was followed.^34^

An initial query was conducted of the pathology database at the University of Pennsylvania for all breast pathology reports from surgical procedures and biopsies signed by a University of Pennsylvania breast pathologist from January 2010 to May 2023 containing the term *metaplastic*. A patient list corresponding to these pathology reports was then generated with duplicate entries removed. The electronic medical record (EMR) was then reviewed for each patient to assess for evidence of germline genetic testing. The following were deemed to be acceptable sources of evidence of germline genetic testing: (1) primary source documentation (ie, a genetic test report from a Clinical Laboratory Improvement Amendments–certified laboratory available within the EMR [preferred]); (2) genetics progress or posttest counseling note; and (3) breast medical oncology progress note containing specifics regarding commercial panel sent, number of genes evaluated, or discussion of medical management in the context of germline variant. Germline testing reports or progress notes were then reviewed to examine whether a PGV was present in a breast cancer susceptibility gene. Genes considered to be associated with breast cancer susceptibility included *BRCA1*, *BRCA2*, *PALB2*, *CDH1*, *TP53*, *PTEN*, *STK11*, *CHEK2* (truncating and missense variants), *ATM*, *RAD51C*, *RAD51D*, *BARD1*, and *NF1.* When clinical genetic testing reports were available, variant classification was based on these reports as pathogenic, likely pathogenic, or of uncertain significance, consistent with American College of Medical Genetics and Genomics and the Association for Molecular Pathology guidelines.^35^ All variants classified as pathogenic and likely pathogenic were considered to be PGVs for the purposes of this study. To confirm up-to-date classification of variants of uncertain significance, all variants of uncertain significance were queried in the ClinVar database, a freely accessible, publicly maintained database of reports of genetic variants and their clinical significance and pathogenicity that incorporates contributions of multiple clinical testing laboratories.^36^ All sources of germline genetic testing were reviewed and confirmed by a medical oncologist with an expertise in germline genetics (P.D.S.) and a genetic counselor (A.A.). Pathology reports for all included participants were reviewed by a breast pathologist (A.N.) to confirm MpBC diagnosis and for subtype classification. Metaplastic carcinomas were subtyped as epithelial-only carcinomas (including low-grade adenosquamous carcinoma, high-grade adenosquamous carcinoma, or pure squamous cell carcinoma), pure monophasic sarcomatoid carcinomas (spindle cell, matrix producing, heterologous), biphasic epithelial and sarcomatoid carcinomas, and low-grade fibromatosis-like metaplastic carcinoma.^11^ The EMR was queried for clinical, demographic, and pathologic data, including age at diagnosis and ER, PR, and ERBB2 status.

### Statistical Analysis

For analysis, descriptive statistics were used to characterize the sample. Two-sided *t* tests were used to compare ages between groups, and, given the small sample sizes, Fisher exact tests were performed to compare proportions. A 2-sided *P* <.05 was considered statistically significant. Exact 95% CIs were calculated for the prevalence of PGVs. The statistical analysis was performed from August 15 to November 23, 2024, using Excel, version 16.76 (Microsoft); Stata, version 18 (StataCorp LLC); and SPSS Statistics, version 30.0.0.0 (IBM) to perform these functions.

## Results

An initial breast pathology database query for reports read at the Hospital of the University of Pennsylvania between 2010 and May 2023 containing the term *metaplastic* yielded 320 pathology reports. A review of the medical records associated with these 320 pathology reports and a breast pathologist review of the pathology reports themselves yielded 272 individuals with a confirmed diagnosis of MpBC. Those excluded were either duplicates (ie, a biopsy and surgical pathology specimen from the same individual) or did not have a confirmed diagnosis of MpBC (including individuals with nonbreast or nonmetaplastic breast tumors).

Of the 272 individuals diagnosed with MpBC, the median age was 58 years (range, 20-102 years). All participants were biological female patients. Complete pathology reports (including hormone receptor and ERBB2 addenda) were available for 264 individuals. Overall, 178 of 264 individuals (67.4%) had triple-negative breast cancer, 75 (28.4%) had ER- and/or PR-positive ERBB2-negative breast cancer, and 11 (4.2%) had ERBB2-positive breast cancer. Of 272 individuals, 143 (52.6%) had documentation of prior germline genetic testing based on criteria described (tested); 129 individuals had no evidence of germline genetic testing on review of the EMR, and these individuals were considered untested. Tested participants were significantly younger than those without testing (median age, 53 years [range, 20-79 years] vs 63 years [range, 29-102 years]; *P* < .001 (Table 1). Of 129 untested individuals, pathology reports for 121 individuals included hormone receptor and ERBB2 status. ER, PR, and ERBB2 status and overall breast cancer subtype did not differ between tested and untested individuals. Those with missing data were excluded from analysis.

**Table 1.** Baseline Characteristics of Study Population^a^

Characteristic	Untested (n = 129 [121 with pathology available])	Tested (n = 143)	*P* value, untested vs tested	PGV in BC gene		*P* value, BC PGV present vs absent
				Absent (n = 119)	Present (n = 24)	
Age at diagnosis, median (range), y	63 (29-102)	53 (20-79)	<.001	55 (20-79)	45 (23-73)	.002
Breast cancer subtype, No. (%)						
Triple-negative (ER and/or PR <1%)	85 (70.2)	93 (65)	.23	76 (63.9)	17 (70.8)	.91
ER and/or PR-positive, ERBB2 negative	29 (24.0)	46 (32.2)		39 (32.8)	7 (29.2)	
ER and/or PR-any, ERBB2 positive	7 (5.8)	4 (2.8)		4 (3.4)	0	
Unknown	8 (6.2)	0				
ER, No. (%)						
Negative (<1%)	102 (84.3)	113 (79)	.42	93 (78.2)	20 (83.3)	>.99
Low (1%-10%)	8 (6.6)	16 (11.2)		14 (11.8)	2 (8.3)	
Positive (>10%-100%)	11 (9.1)	14 (9.8)		12 (10)	2 (8.3)	
Unknown	8 (6.2)	0				
PR, No. (%)						
Negative (<1%)	99 (81.8)	115 (80.4)	.89	95 (79.8)	20 (83.3)	.92
Low (1%-10%)	12 (9.9)	17 (11.9)		14 (11.8)	3 (12.5)	
Positive (>10%-100%)	10 (8.3)	11 (7.7)		10 (8.4)	1 (4.2)	
Unknown	8 (6.2)	0				
ERBB2 status, No. (%)						
Nonamplified	114 (94.2)	139 (97.2)	.36	115 (96.6)	24 (100)	>.99
Amplified	7 (5.8)	4 (2.8)		4 (3.4)	0	
Unknown	8 (6.2)	0		0	0	
Metaplastic subtype, No. (%)						
Biphasic (epithelial and sarcomatoid)	NA	75 (52.4)	NA	65 (54.6)	10 (41.7)	.31
Epithelial		44 (30.8)		35 (29.5)	9 (37.5)	
Pure sarcomatoid		19 (13.3)		16 (13.4)	3 (12.5)	
Fibromatosis-like		0		0	0	
Unknown		5 (3.5		3 (2.5)	2 (8.3)	
Germline test results, No. (%)						
PGV in a breast cancer gene	NA	24 (16.8)	NA	0	24 (100)	NA
* gBRCA1*		17 (11.9)		0	17 (70.8)	
* gBRCA2*		5 (3.5)		0	5 (20.8)	
* gPALB2*		1 (0.7)		0	1 (4.2)	
* gCHEK2*		1 (0.7)		0	1 (4.2)	
* gMUTYH*		1 (0.7)		1 (0.8)	0	
* gHOXB13*		1 (0.7)		1 (0.8)	0	
* gCDKN2A*		1 (0.7)		1 (0.8)	0	
* gSDHA*		1 (0.7)		1 (0.8)	0	
Variants of uncertain significance		31 (21.7)		29 (24.4)	2 (8.3)	

Abbreviations: BC, breast cancer; ER, estrogen receptor; ERBB2, erb-b2 receptor tyrosine kinase 2; NA, not applicable; PGV, pathogenic germline variant; PR, progesterone receptor.

^a^ Two-sided *t* tests were used to compare ages. Fisher exact tests were used to compare proportions. Analyses were performed among those with complete data.

Germline testing result reports were available for review for 112 of 143 tested individuals (78.3%), and the remainder had germline test result documentation in genetics or oncology progress notes (Table 2). Details specifying the testing type or platform (single site, *BRCA1/2* only, multigene panel) were available for 125 of the 143 tested individuals, including the 112 individuals with available reports as well as 13 of those with genetics or oncology progress notes indicating germline mutations (Table 2). Most individuals (75.2% [94 of 125]) had multigene panel testing, and, of these, most had more than 15 genes evaluated. The remaining participants had testing for *BRCA1* and *BRCA2* only except for 1 individual who had single-site testing in the context of a known familial variant.

**Table 2.** Details of Germline Genetic Testing Including the Source of Documentation and Number of Genes Evaluated

Characteristic	No. (%)
Genetic testing confirmation (n = 143)	
Test report	112 (78.3)
Genetics or oncology progress note	31 (21.7)
Data available on specific panel or genes evaluated	13 (41.9)
Testing type or platform (n = 125)	
Single site	1 (0.8)
*BRCA1* and *BRCA2* only	30 (24)
Multigene panel test	94 (75.2)
With ≤15 genes included	21 (16.8)
With >15 genes included	73 (58.4)

Twenty-four individuals (16.8% [95% CI, 11.1%-23.9%]) with germline testing had PGVs in breast cancer susceptibility genes, including *BRCA1* (n = 17 [70.8%]), *BRCA2* (n = 5 [20.8%]), *PALB2* (n = 1 [4.2%]), and *CHEK2* (n = 1 [4.2%]). This corresponds to a *BRCA1* prevalence of 11.9% (n = 17), a *BRCA2* prevalence of 3.5% (n = 5), a *PALB2* prevalence of 0.7% (n = 1), and a *CHEK2* prevalence of 0.7% (n = 1) among the 143 individuals who were tested. Almost all known PGVs were unique; only 2 patients bore the same Polish founder variant (*BRCA1* c.181T>G [p.C61G]) (Table 3). Pathogenic variants in genes not typically associated with breast cancer were also present in 4 patients. These included PGVs in *MUTYH*, *HOXB13*, *CDKN2A*, and *SDHA*. Variants of uncertain significance were found in 31 individuals.

**Table 3.** Characteristics of PGV Carriers

Gene containing PGV and DNA sequence change	Protein sequence change	Molecular consequence	Metaplastic subtype	Age, y	Breast cancer subtype
*BRCA1*					
c.3254_3255dup	p.Leu1086fs	Frameshift	Biphasic	40s	TNBC
c.4183C>T	p.Gln1395Ter	Missense	Biphasic	30s	ER positive or PR negative, ERBB2 negative
c.3481_3491del11	p.Glu1161fs	Frameshift	Biphasic	50s	TNBC
c.117_118del	p.Cys39_Asp40delinsTer	Frameshift nonsense	Biphasic	30s	ER negative or PR positive, ERBB2 negative
c.3748G>T	p.Glu1250Ter	Nonsense	Biphasic	70s	TNBC
c.5266dup	p.Gln1756fs	Frameshift	Epithelial	30s	ER positive or PR negative, ERBB2 negative
c.4986 + 4A>G	NA	Intron variant	Epithelial	50s	TNBC
c.181T>G	p.Cys61Gly	Missense	Epithelial	70s	TNBC
c.181T>G	p.Cys61Gly	Missense	Unknown	20s	ER negative or PR positive, ERBB2 negative
c.676del	p.Cys226fs	Frameshift	Unknown	40s	TNBC
del (Exon 7-8)	NA	Frameshift	Pure sarcomatoid	20s	TNBC
Unknown	Unknown	NA	Pure sarcomatoid	60s	TNBC
Unknown	Unknown	NA	Pure sarcomatoid	40s	TNBC
Unknown	Unknown	NA	Biphasic	30s	TNBC
Unknown	Unknown	NA	Biphasic	60s	TNBC
Unknown	Unknown	NA	Biphasic	40s	TNBC
Unknown	Unknown	NA	Biphasic	30s	TNBC
*BRCA2*					
c.4131_4132insTGAGGA	p.Thr1378Ter	Nonsense	Biphasic	30s	ER negative or PR positive, ERBB2 negative
c.1909 + 1G>A	NA	Splice donor	Epithelial	40s	ER positive or PR negative, ERBB2 negative
c.4631delA	p.Asn1544fs	Frameshift	Epithelial	40s	TNBC
c.8487 + 1G>A	NA	Splice donor	Epithelial	30s	TNBC
c.8177A>G	p.Tyr2726Cys	Missense	Epithelial	50s	TNBC
*PALB2*					
Unknown	Unknown	NA	Epithelial	40s	ER positive or PR positive, ERBB2 negative
*CHEK2*					
c.1555C>T	p.Arg519Ter	Nonsense	Epithelial	40s	TNBC

Abbreviations: ER, estrogen receptor; ERBB2, erb-b2 receptor tyrosine kinase 2; NA, not applicable; PGV, pathogenic germline variant; PR, progesterone receptor; TNBC, triple-negative breast cancer.

Age at diagnosis, metaplastic subtype, and biomarker (ER, PR, ERBB2) status were evaluated in those with or without PGVs (Table 1). The median age at breast cancer diagnosis was significantly lower for those with vs without PGVs (45 years [range, 23-73 years] vs 55 years (range, 20-79 years]; *P* = .002). Metaplastic subtype and biomarker status did not differ between those with and those without PGVs (Table 2). Approximately two-thirds of the population had triple-negative breast cancer, defined as ER less than 1%, PR less than 1%, and ERBB2 nonamplified (immunohistochemistry subtype 0, 1, or 2 with negative in situ hybridization), whereas one-third had hormone receptor–positive breast cancer and 4 individuals had ERBB2-amplified MpBC. All patients with PGVs either received a diagnosis at younger than 65 years and/or had triple-negative breast cancer (Table 3).

The histologic subtype of most (52.4% [75 of 143]) of the MpBCs was biphasic (epithelial and sarcomatoid), followed by epithelial (30.8% [44 of 143]) and pure sarcomatoid (13.3% [19 of 143]). There was no significant difference in the distribution of histologic subtypes between those with and those without PGVs (*P* = .39). Among those with PGVs in *BRCA1*, the biphasic, epithelial, and pure sarcomatoid subtypes were all represented. Almost all cases with PGVs in *BRCA2*, *PALB2*, and *CHEK2* were of the epithelial subtype, although these PGVs were fewer in number (Table 3). Overall, there was no clear association between PGVs in any breast cancer susceptibility gene and a specific histologic subtype of MpBC.

## Discussion

We present clinical germline genetic testing results from 143 patients pathologically proven to have MpBC at our institution over a 13-year period of time. To our knowledge, this cohort represents the largest series to date of patients with MpBC analyzed for PGVs in breast cancer susceptibility genes. The study population was ascertained from a pathology database rather than from a genetics or clinical database to minimize bias and included patients regardless of age at breast cancer diagnosis, hormone receptor positivity, or ERBB2 amplification status. All pathology reports underwent repeated review by a breast pathologist, and all germline genetic testing reports underwent review by genetics providers. We found that 16.8% (95% CI, 11.1%-23.9%) of participants with MpBC who underwent germline testing had PGVs in breast cancer susceptibility genes, with PGVs seen across MpBC subtypes and with most PGVs occurring in *BRCA1* (11.9% variant prevalence) and *BRCA2* (3.5%).

The observed PGV prevalence is higher than the 5.03% frequency noted in a large, population-based study evaluating PGV prevalence in an unselected population of patients with breast cancer.^37^ This variant prevalence also exceeds or is comparable to the 7.8% to 13.2% breast cancer susceptibility gene PGV prevalence seen in other studies of patients referred for clinical genetic testing.^38,39,40,41^ Furthermore, the variant prevalence of 16.8% noted in the present study of patients with MpBC is relatively high in comparison with the PGV frequency estimates in patients with triple-negative breast cancer (10.6%-15.8%).^27,28,29,42,43,44^ The highest cited frequency of PGVs in individuals with triple-negative breast cancer was from a study in which the median age of participants was 40 years,^28^ whereas the median age of individuals included in the present analysis was 53 years (range, 20-79 years). Although formal cross-study comparisons are not possible, and although the 95% CI of our observed 16.8% PGV prevalence overlaps with prior studies, even the lower limit of this 95% CI suggests that a substantial proportion of these patients with MpBC have PGVs. Indeed, to our knowledge, the 16.8% PGV prevalence among patients with MpBC represents the highest variant frequency in any reported histologic subtype-specific population of patients with breast cancer, although not all those with metaplastic carcinoma were referred for clinical testing.

Most PGVs seen in the present study were in *BRCA1* (11.9% PGV prevalence, comprising 71% of all breast cancer susceptibility PGVs seen). An association between MpBC and *BRCA1* has previously been suggested in case report form^20,21,22,23,24,25^ and in 2 small series.^26,45^ Rodríguez-Fernández et al^45^ evaluated the histology of 93 patients with breast cancer and known germline variants in *BRCA1* or *BRCA2*; 3.2% of known affected carriers had metaplastic carcinoma compared with 0.8% (25 of 3157) of a genetically unselected reference population. Corso and colleagues^26^ reported on a series of 23 individuals who were referred for and received genetic testing and who were found to have MpBC. They noted PGVs in breast cancer susceptibility genes in 14 of 23 individuals (60.9%), including in *BRCA1* (n = 13) and *TP53* (n = 1). Unlike the present report, these studies ascertained patients on the basis of a known breast cancer gene PGV or based on referral to genetics providers, indicating an enrichment of individuals with suspected variants; indeed, 2 individuals in the latter report were referred to genetics due to known familial *BRCA1* PGVs. An association between MpBC and *NF1* has also been discussed in case report form,^46^ although no *NF1* variants were seen in the present report. No PGVs were seen in the breast cancer susceptibility genes *RAD51C*, *RAD51D*, or *BARD1*, all of which are known to be associated with triple-negative breast cancer,^47,48^ although not all patients had multigene panel tests including these genes.

Current guidelines, including those published by the National Comprehensive Cancer Network (NCCN),^49^ the American Society of Clinical Oncology–Society of Surgical Oncology (ASCO-SSO) panel,^50^ and the American Society of Breast Surgeons,^51^ advocate for germline testing for patients with breast cancer with relatively broad parameters defining eligible populations. The only histopathology-specific considerations included in these guidelines are for patients with triple-negative breast cancer; both NCCN and ASCO-SSO guidelines recommend germline testing for patients of any age with this diagnosis. Outside of this subtype, ASCO-SSO guidelines also recommend testing for individuals who received a diagnosis of breast cancer at or younger than 65 years, and NCCN guidelines recommend testing for individuals who received a diagnosis at or younger than 50 years. In the present analysis, patients found to have breast cancer–associated PGVs included 7 (29.2%) with hormone receptor–positive disease as well as 2 (8.3%) older than 65 years. However, the 7 patients with hormone receptor–positive disease and PGVs received a diagnosis between 29 and 45 years of age, and those who received a diagnosis at older than 50 years had triple-negative breast cancer; therefore, these individuals would have been captured for germline testing eligibility by current guidelines. Despite this, given the high prevalence of clinically meaningful PGVs seen in the present cohort and the rarity of patients with MpBC, the present report suggests that regardless of age at diagnosis, hormone receptor status, or metaplastic subtype, germline genetic testing for all patients with MpBC may be high yield.

### Strengths and Limitations

Strengths of this study include a relatively substantial sample size for this rare subtype of breast cancer; ascertainment of study participants from a pathology database rather than from a genetics database; population unselected for hormone receptor or ERBB2 status; pathologic confirmation of MpBC by breast-specific pathologists rather than identification of MpBC based on *International Classification of Diseases* codes, which can pose a challenge for classification of rare subtypes. Additional strengths include primary source documentation of germline genetic testing results for most included participants and completion of germline genetic testing in a clinical setting.

This study also has important limitations. Although the participants included in this study were not overtly selected based on age, hormone receptor positivity, or ERBB2 expression, patients who underwent testing were significantly younger at diagnosis than those who did not. This observation reflects referral for clinical germline genetic testing prompted by young age at diagnosis concordant with NCCN guidelines, which for several years recommended genetic testing referral for breast cancer diagnosis at or younger than 45 years.^52,53^ Younger age at diagnosis of tested patients may have enriched for carriers of PGVs. Two other potential differences between tested and untested populations may be (1) proportion of patients with Ashkenazi Jewish ancestry and (2) family history of cancer. We were unable to reliably obtain these data, although only 1 of the PGVs identified represents an Ashkenazi Jewish founder mutation (Table 3). Furthermore, the study was conducted at an academic center that not only is a breast oncology referral center but has a dedicated genetics referral center, which may have augmented our observed prevalence by increasing the number of young patients with a family history included in our study population. This may have resulted in higher rates of PGVs in this population and may affect the external validity of our findings. A sensitivity analysis stratified by age would be an informative next step in a future larger study.

## Conclusion

In the largest investigation to date of germline genetic testing for patients with MpBC, this cohort study found a 16.8% prevalence of clinically meaningful PGVs in breast cancer susceptibility genes, mostly in *BRCA1*. This represents a substantial population of patients with MpBC found to have PGVs. Recognizing that the present study was conducted at a single academic oncology referral center with an embedded genetics program, these findings warrant external validation with larger sample sizes and diverse clinical settings. Current guidelines likely encompass most patients with MpBC who are often young and/or have triple-negative disease. However, because of the rarity of MpBC, the expanding therapeutic implications for affected individuals, and the potential effect on risk mitigation strategies for unaffected family members while external validation studies are ongoing, all patients with MpBC regardless of hormone receptor status or age at diagnosis may warrant consideration for genetic testing.

## Notes

Supplement.
Data Sharing Statement
