# Supplementary material for: Pathogenic Germline Variants in Patients With Metaplastic Breast Cancer
Source: JAMA Netw Open. 2025 Feb 18;8(2):e2460312. doi: 10.1001/jamanetworkopen.2024.60312 (PMC11836754; doi:10.1001/jamanetworkopen.2024.60312)
Supplement: Supplement. — Data Sharing Statement [file jamanetwopen-e2460312-s001.pdf]

Data Sharing Statement

Demarest. Pathogenic Germline Variants in Patients With Metaplastic Breast Cancer. *JAMA Netw Open*. Published February 18, 2025. doi:10.1001/jamanetworkopen.2024.60312

Data
Data available: Yes
Data types: Deidentified participant data
How to access data: Deidentified participant data will be made available upon request as allowed by our institutional review board policies.
When available: With publication

Supporting Documents
Document types: None

Additional Information
Who can access the data: All requests will be considered as allowed by our institutional review board and scientific board policies.
Types of analyses: For inclusion in larger datasets.
Mechanisms of data availability: With investigator support after approval of a proposal and with signed data access agreements as required.
